# Control of chemical reactivity by transition-state and beyond

**DOI:** 10.1039/c6sc01066k

**Published:** 2016-04-13

**Authors:** Hua Guo, Kopin Liu

**Affiliations:** a Department of Chemistry and Chemical Biology, University of New Mexico Albuquerque New Mexico 87131 USA hguo@unm.edu; b Institute of Atomic and Molecular Sciences, Academia Sinica Taipei 10617 Taiwan kliu@pub.iams.sinica.edu.tw; c Department of Physics, National Taiwan University Taipei 10617 Taiwan

## Abstract

It has been long established that the transition state for an activated reaction controls the overall reactivity, serving as the bottleneck for reaction flux. However, the role of the transition state in regulating quantum state resolved reactivity has only been addressed more recently, thanks to advances in both experimental and theoretical techniques. In this perspective, we discuss some recent advances in understanding mode-specific reaction dynamics in bimolecular reactions, mainly focusing on the X + H_2_O/CH_4_ (X = H, F, Cl, and O(^3^P)) systems, extensively studied in our groups. These advances shed valuable light on the importance of the transition state in mode-specific and steric dynamics of these prototypical reactions. It is shown that many mode-specific phenomena can be understood in terms of a transition-state based model, which assumes in the sudden limit that the ability of a reactant mode for promoting the reaction stems from its coupling with the reaction coordinate at the transition state. Yet, in some cases the long-range anisotropic interactions in the entrance (or exit) valley, which govern how the trajectories reach (or leave) the transition state, also come into play, thus modifying the reactive outcomes.

## Abstract 2

State-specific reaction dynamics is largely controlled by transition state.

## Graphical abstract

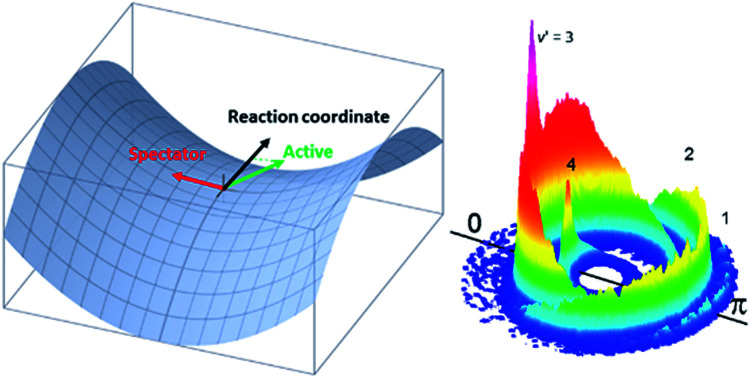

## I. Introduction

The textbook description of a chemical reaction typically invokes the concept of a transition state, through which the transformation of reactants to products is transpired. The transition state was first proposed by Eyring, Wigner, Evans, and Polanyi as an activated complex in a reaction, which, once reached, dissociates irreversibly to the products.^1–3^ As a result, it serves as a bottleneck for the reaction flux, thus controlling reaction kinetics. This conceptual construct is not only very useful for understanding chemical reactivity, but also forms the basis of the powerful transition-state theory (TST).^1–3^ TST is based on the premise that the rate coefficient of a bimolecular reaction is proportional to the reaction flux passing through a dividing surface that separates the reactants from products.^4^ A convenient location of the dividing surface is at the top of the free-energy barrier, in which the recrossing is minimal. It should however be noted that the classical TST is not quantitatively accurate. Quantum mechanical effects, such as energy quantization and tunneling can be taken into account using semi-classical approximations.^5^ Nowadays, the validity and applicability of TST are firmly established.

Unfortunately, the precise location of the transition state cannot be readily defined. Even for the same reaction, the position of the free-energy barrier might change with temperature. Indeed, the most sophisticated version of TST is variational, which minimizes the rate coefficients to account for recrossing.^5^ Experimental detection of the transition state is also difficult because of the ultrashort lifetime of the activated complex.^6^ To better understand the transition state and its influence on reaction kinetics and dynamics, it is thus best approaching the problem from a theoretical perspective, from which the reaction is simply the result of nuclear motion on a potential energy surface (PES).^4,7^ The PES is the sum of the nuclear repulsion and electronic energy within the Born–Oppenheimer or adiabatic approximation, which separates the nuclear motion from the electronic one based on the large mass disparity between the two. For most reactions occurring at low temperature, only the ground electronic state is involved. Once the PES is known, the reaction dynamics (and equivalently ro-vibrational spectroscopy) can be characterized, at least in principle, by solving the nuclear Schrödinger equation. In this context, spectroscopy and scattering can be regarded as experimental ways to probe the PES.

For a typical activated bimolecular reaction, there is always a saddle point on the PES that is the highest point along the reaction coordinate. This stationary point defines the topographic features nearby in the transition-state region for the reaction. Specifically, a first-order saddle point features one coordinate associated with a negative second derivative while all other coordinates possess positive second derivatives. The vector for the normal mode with an imaginary frequency represents the reaction coordinate that traverses the barrier.^4^ In TST, the dividing surface is often defined at the saddle point perpendicular to the reaction coordinate.^5^

It is now well established that the transition-state controls the overall reactivity, which underscores the success of TST in predicting the rate coefficients. There is also an increasing body of evidence that suggests that the transition state also plays an important role in regulating reaction dynamics.^8–12^ A most common manifestation of the transition-state control of reaction dynamics is mode specificity, namely the differing ability of various reactant modes in promoting the reaction.^13,14^ As described below, technological advances have enabled one to prepare the reactant in a single quantum state, and the measurement of its reactivity shed valuable light on how effective it helps to overcome the reaction barrier.^15^ An equally informative approach is to probe the product energy disposal with quantum resolution, which can now be achieved with exquisite detail with various laser and product imaging techniques,^16–19^ also discussed below. In the same time, new developments in quantum reactive scattering methods and PES construction have also advanced to a level that detailed comparison with state-to-state experimental data becomes possible.^11,20–24^ The accumulation of detailed quantum-state resolved data allows a much more detailed understanding of the transition-state control of the reaction dynamics.

Of course, reaction dynamics can also be affected by other factors. One important quantum mechanical factor is resonances, which are metastable states formed on the PES, many near the transition state. These states can impact reaction dynamics in a significant way, as demonstrated by ample experimental^16,25,26^ and theoretical evidence.^11,27^ However, discussion of the impact of resonances is beyond the scope of this review.

In this perspective, we discuss recent advances in our understanding of transition-state control of reaction dynamics, from both experimental and theoretical perspectives. While such control is indeed dominant, an important point that is becoming widely recognized is that this ideal textbook description of reactions requires some refinements, because of important and prevalent features on the PES. For example, shallow wells in the entrance channel could exert sufficient forces on the approaching reactant molecules, leading to modifications of the transition-state control of the reactivity and dynamics. By the same token, potential wells in the exit channel may also exercise influence on the product energy disposal. On the other hand, the anisotropy of the PES suggests that the reactivity depends on the approach of the reactants relative to each other, as some preferred angles of approach may avoid unproductive collisions. A better understanding of these factors will not only deepen our understanding of reaction dynamics, but also potentially allow control of reaction yield and product branching.

## II. Transition state and its control of reaction dynamics

As alluded to above, an important manifestation of transition-state control of reaction dynamics is mode specificity.^12,13^ The most celebrated model to rationalize mode specificity is the Polanyi rules, which were distilled from studies of atom–diatom reactions with various reaction energies and barrier locations.^8^ For reactions with a reactant-like, or early, barrier, relative translation energy between the two collision partners is the most effective in overcoming the barrier. On the other hand, for reactions with a product-like, or late, barrier, vibrational excitation has a higher efficacy in enhancing the reactivity than the translational energy. Polanyi's model, which emphasizes the importance of the location of the transition state in enhancing reactivity for activated reactions, has been widely used for predicting the relative efficacy of vibrational and translational excitations in promoting the reaction.

While intuitive and insightful, the Polanyi rules only offer qualitative predictions because the earliness/lateness of the barrier location is difficult to quantify. More importantly, they do not, strictly speaking, apply to reactions involving polyatomic reactants. As a result, these empirical rules provide little guidance on the relative efficacies of the internal, namely vibrational and rotational, modes of the reactants in promoting the reaction. Recently, the Polanyi rules have been extended to polyatomic reactions by examining the coupling of reactant modes with the reaction coordinate at the transition state.^28,29^ In this Sudden Vector Projection (SVP) model, the time scale of the collision is assumed to be much shorter than that required for intramolecular vibrational energy redistribution (IVR) in the reactants, a condition that is generally satisfied for the reactions discussed in this perspective. As a result, the reaction can be considered in the sudden limit, in which the energy deposited into a particular reactant mode is preserved until the transition state is reached. In this sudden limit, the coupling between a reactant mode and the reaction coordinate at the transition state is approximated by the projection of the corresponding normal mode vectors. If the projection is large, the reactant mode is strongly coupled with the reaction coordinate. In this case, energy deposited into this mode flows readily into the reaction coordinate, thus enhancing the reactivity. On the other hand, if the projection is small, the coupling with the reaction coordinate is weak, and the energy flow is limited. This leads to a low ability to enhance the reactivity.

The SVP model is thus formally quantitative and treats all reactant modes on an equal footing. Like the Polanyi rules, the SVP model emphasizes the importance of the transition state, represented by the saddle point of the underlying PES, in mode specificity. However, the latter uses the projection of reactant mode on the reaction coordinate at the transition state, rather than the location of the barrier, as the descriptor of the coupling strength. It has been shown that the coupling strength is closely correlated with the position of the barrier and the predictions of the SVP model are completely consistent with the Polanyi rules for atom–diatomic reactions.^28^

By invoking microscopic reversibility, the mode specificity of the reverse reaction dictates how energy is disposed in the product modes of the forward reaction. Thus, the SVP model can also be used to predict product energy disposal.^28,29^ In the same spirit, a product mode that has a large projection on to the reaction coordinate at the transition state is expected to be excited, and *vice versa*.

It should be noted that the idea of attributing mode specificity to coupling with the reaction coordinate at the transition sate has been percolating in the reaction dynamics community for some time. For example, Schatz and Ross have proposed a Franck–Condon model to approximate reactive scattering using inelastic scattering wavefunctions.^30^ Franck–Condon models have also been widely used to predict product state distributions in reactions.^31–33^ More recently, Skodje and coworkers have proposed a Franck–Condon model to approximately compute the reactive *S*-matrix elements,^34^ and Manthe and coworkers devised an exact scheme for state-to-state reactive scattering based on transition state wave packets.^35^ The SVP model can be considered as a simplified sudden model, in which the Franck–Condon factors are approximated by projections of normal mode vectors onto the reaction coordinate vector at the transition state.^28^ Its strength is its simplicity, as it requires neither PESs nor dynamical calculations. Only properties of the transition state are needed. Similar ideas of projecting reactant vectors to reaction coordinate at the transition state have been put forth by Wang and Bowman^36^ and by Siebrand *et al.*^37^ for treating mode-specific tunneling.

The SVP model has been applied to several prototypical reactions and in most cases its predictions have been borne out.^9,38^ The general success of this transition-state based model attests the importance of the transition state in controlling reaction dynamics. In particular, the SVP model correctly predicts the mode specificity in some reactions for which a naïve extension of Polanyi rules failed, some of which are discussed in more details below. However, it is expected that the SVP model will fail if the reaction is not sudden, in which the IVR may be prevalent. This limit is more appropriately described with an adiabatic model, such as the one based on reaction path Hamiltonian.^39^

## III. Experimental and theoretical advances

With the advent of tunable infrared (IR) lasers, two molecular beam approaches have been developed to investigate the impact of the vibrational excitation of reactants on chemical reactions under single-collision conditions. The PHOTOLOC method pioneered by Zare's group^40^ employs a co-expansion of a gas mixture. A pulsed nanosecond laser initiates the reaction by photolyzing one of the gaseous molecules (the precursor) to generate the reactive radicals (typically the Cl or H atoms). Another IR laser is employed to prepare the vibrational excited reactants. A third probe laser is fired tens of nanosecond later to interrogate the internal states of reaction products *via* the resonance-enhanced multiphoton ionization (REMPI) scheme. A velocity-sensitive time-of-flight (TOF) spectrometer then registers the temporal profile of the desired ions, which can be converted to the speed distribution of the probed product state, assuming a negligible change of the recoil velocity of the REMPI-ions from the neutral products. The measured speed distribution represents a one-dimensional (1D) projection of the desired three-dimensional (3D) state-tagged angular distribution, which can be recovered by the law of cosines.^40^

The crossed molecular beam approach, in particular that equipped with a time-sliced velocity map imaging detector,^41^ offers a powerful alternative. The velocity-map imaging technique takes advantage of multiplex detection,^42,43^ and the time-sliced version^41,44,45^ not only yields higher resolution to reveal the correlated state distributions of the coproducts from the raw image,^18,46,47^ but also alleviates much ambiguity in analyzing an (intrinsically) cylindrically asymmetric image by simplifying the density-to-flux correction^48^ from a 3D problem (for a conventional crushed image) to a 2D one.^41^ The effect of vibrational excitation on reactivity has also been investigated using a crossed-beam machine with the traditional universal mass spectroscopic detection method.^49^

Both PHOTOLOC and crossed-beam approaches could be operated in the TOF mass-spectrometric mode by scanning the probe laser wavelength to obtain the integral cross section (ICS) information on product channel branching ratios and state distributions. To obtain the vibrational enhancement factor, *i.e.*, the relative reactivity *σ*^#^/*σ*_0_ of vibrationally excited (denoted by the superscript “#”) *versus* ground-state (the subscript “0”) reactants, one needs to determine the IR pumping efficiency (*n*^#^/*n*_0_).^50–52^ For illustration, the product signal from the ground-state reaction (*i.e.*, IR off) can be expressed as

1 *S*^off^ = *cn*_0_*σ*_0_.

As the IR laser is on, the signals become

2 *S*^on^ = *c*[(*n*_0_ − *n*^#^)*σ*_0_ + *n*^#^*σ*^#^],

where *c* is a proportional constant, *n*_0_ and *n*^#^ denote the concentrations of the ground state (IR-off) and laser excited reactants, respectively, and *σ*_0_ and *σ*^#^ are the corresponding reaction cross sections. Eqn (1) and (2) then lead to

3 *S*^on^/*S*^off^ = 1 − (*n*^#^/*n*_0_)(1 − *σ*^#^/*σ*_0_).

Clearly, knowledge of *n*^#^/*n*_0_ is required in order to derive the desired quantity of *σ*^#^/*σ*_0_ at a given collisional energy. It is instructive to note that the measured ratio of *S*^on^/*S*^off^ gives an immediate impression about relative values of *σ*^#^ to *σ*_0_: (i) if *S*^on^/*S*^off^ > 1 (*i.e.*, signal enhancement upon IR irradiation), then *σ*^#^ > *σ*_0_, (ii) *S*^on^ ∼ *S*^off^ (*i.e.*, signal strengths unchanged) implies *σ*^#^ ∼ *σ*_0_, and (iii) *S*^on^/*S*^off^ < 1 (*i.e.*, signal depletion) yields *σ*^#^ < *σ*_0_.

**Fig. 1:**
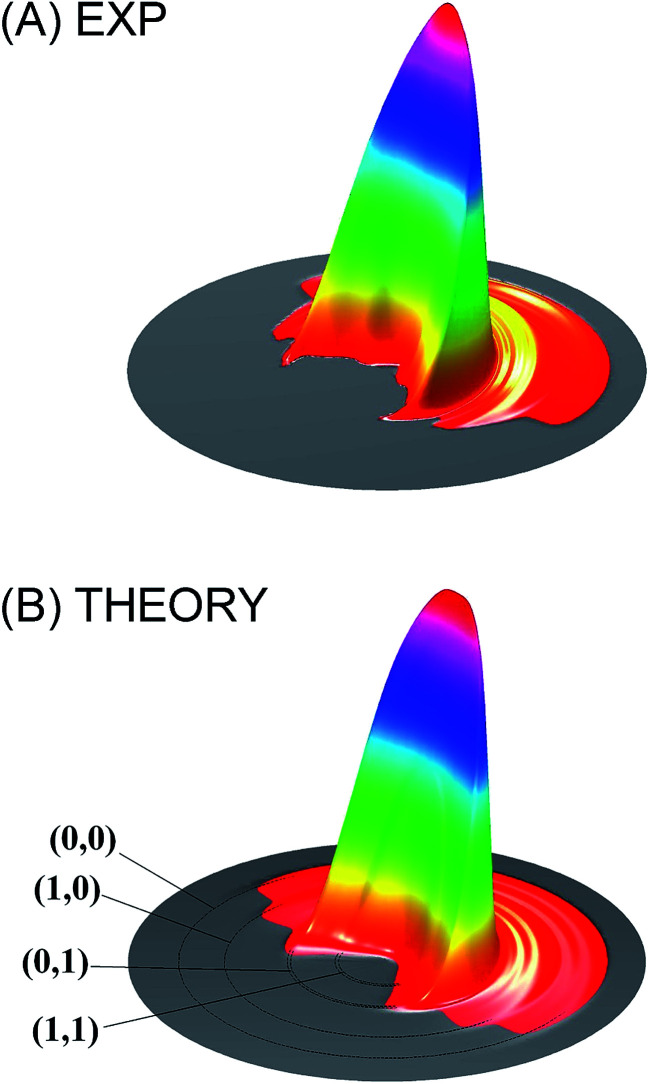
Comparison of the measured differential cross section (A) with that obtained from full-dimensional quantum dynamical calculations (B) for the HD + OH → H_2_O + D reaction at the collision energy of 6.9 kcal mol^−1^. Adapted from ref. 54 with permission.

Theoretically, significant progress has also been made in three areas. The first has to do with the emergence of highly accurate electronic structure theories such as multi-reference configuration interaction (MRCI) and coupled cluster (CC) methods. These methods, particularly in the explicit correlated (F12) version, can now provide chemically accurate (<1 kcal mol^−1^) energies with reasonable computational costs.^53^ The second advance is the efficient and accurate analytical representation of PESs from these electronic structure calculations. It is now possible to develop globally accurate high-dimensional PESs for reactive systems.^11,23,24^ Finally, quantum reactive scattering on these accurate PESs can now be performed for tetra-atomic systems, some with state-to-state resolution. These advances allow a direct comparison with experimental measurements, as in the recent study of the HD + OH → H_2_O + D reaction. In this exemplary case, the calculated and measured product angular distributions, namely the differential cross sections (DCSs), of this four-atom reaction are compared.^54^ As shown in Fig. 1, the agreement is excellent. Thanks to these developments, quantum reactive scattering has moved beyond the atom–diatom reactions. For a detailed discussion of the recent advances in quantum reactive scattering, the reader is referred to the recent review on this topic.^11^

## IV. Case studies

### A. X + H_2_O ↔ HX + OH reactions

**Fig. 2:**
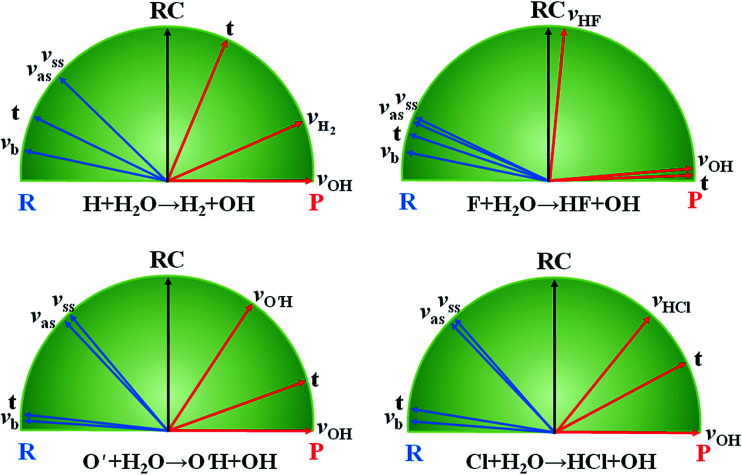
SVP projections of reactant (R) vectors and product (P) vectors onto the reaction coordinate (RC) at the transition state for the three reactions. Adapted from ref. 29 with permission.

The hydrogen abstraction of water by H/Cl has served as a prototypical model to understand the transition-state control of reactivity. Earlier theoretical studies have predicted that the stretching vibrations of the H_2_O reactant greatly enhance the reactivity,^55^ which was confirmed by later experiments.^56–59^ These two endothermic reactions have late barriers, consistent with Hammond's postulate,^60^ which states that in a series of reactions, the more endothermic (exothermic) the reaction, the more the transition state will resemble the products (reactants). As a result, the strong vibrational enhancement of reactivity can be rationalized by the Polanyi rules.^8^ In Fig. 2, projections of various reactant modes onto the reaction coordinate at the transition state are shown for the two reactions, which illustrate that this strong mode specificity is also predicted by the SVP model.^29^ Interestingly, both the symmetric and antisymmetric stretching modes of H_2_O have roughly the same efficacies, as predicted by the SVP model (Fig. 2). As discussed latter, this is due to the local-mode nature of the stretching vibrational modes of H_2_O.^61^ The observed mode specificity has later been reproduced by exact quantum dynamics (QD) studies.^62–64^ An interesting corollary of mode specificity is the so-called bond selectivity. For example, HOD in which the OH or OD vibration was excited was found to react by preferential cleavage of the excited bond.^65,66^ Obviously, the mass difference between H and D renders the OH and OD local modes uncoupled, and the bond cleavage proceeds *via* the corresponding transition state on the PES. The bond selectivity is reproduced by QD calculations,^67–70^ and well understood in terms of the SVP model.^29^

While the mode specificity and bond selectivity of the H/Cl + H_2_O/HOD reactions are consistent with Polanyi's predictions, the situation is quite different for the F + H_2_O reaction. In contrast to the former two reactions that have late barriers, the latter has a low and early barrier. The Polanyi rules thus predict that the reaction would be enhanced more effectively by the translational energy. However, our QD calculations based on an *ab initio* based PES indicated that except for low collision energies all three vibrational modes of H_2_O enhance the reaction more effectively than translation.^71^ This apparent inconsistency was resolved by the SVP model, as shown in Fig. 2, which found that while the couplings between the vibrational modes and the reaction coordinate at the transition state are weak, they are stronger than that for the translational mode.^29^ The inability of the Polanyi rules to predict the mode specificity in this and other polyatomic reactions^72^ suggests that the location of the barrier may not necessarily be the best descriptor of mode specificity. Rather, the coupling between the reactant mode and reaction coordinate at the transition state, as suggested by the SVP model, is perhaps a more reliable way to predict mode specificity. The SVP model also correctly predicted the strong vibrational excitation in the HF product and the spectator nature of the OH moiety in this reaction, in good agreement with both experiment^73^ and theory.^74,75^ The SVP values for the HF and OH vibrational modes are close to the extremes (∼1.0 and ∼0.0), underlying their respective coupling strengths with the reaction coordinate at the transition state.^29^

More detailed evidence for transition-state control of reaction dynamics can be found at the state-to-state level. One of the interesting observations is that the OD product in the H + D_2_O reaction is mostly found in its ground vibrational state, even when the stretching vibrations of D_2_O is excited with one stretching quantum.^58^ This is termed the “memory loss” effect, as the product distributions appear to be independent of the reactant excitation.^76^ On one hand, it suggests that the non-reactive OD bond is a spectator in the reaction. Perhaps more interestingly, this “memory loss” effect should be considered in the local-mode picture in which the D_2_O normal-mode vibrations is expressed as a linear combination of symmetrized local-mode basis.^61^ In this local-mode picture, the cleavage of a particular OD bond almost exclusively proceeds *via* one of the two equivalent transition states on the PES where this OD bond is elongated.^77^ As a result, the other non-reactive OD bond retains its vibrational ground state in the product. This is exactly the same reason that the two stretching modes of H_2_O promote the H + H_2_O reaction with roughly the same efficacy, as alluded above.

**Fig. 3:**
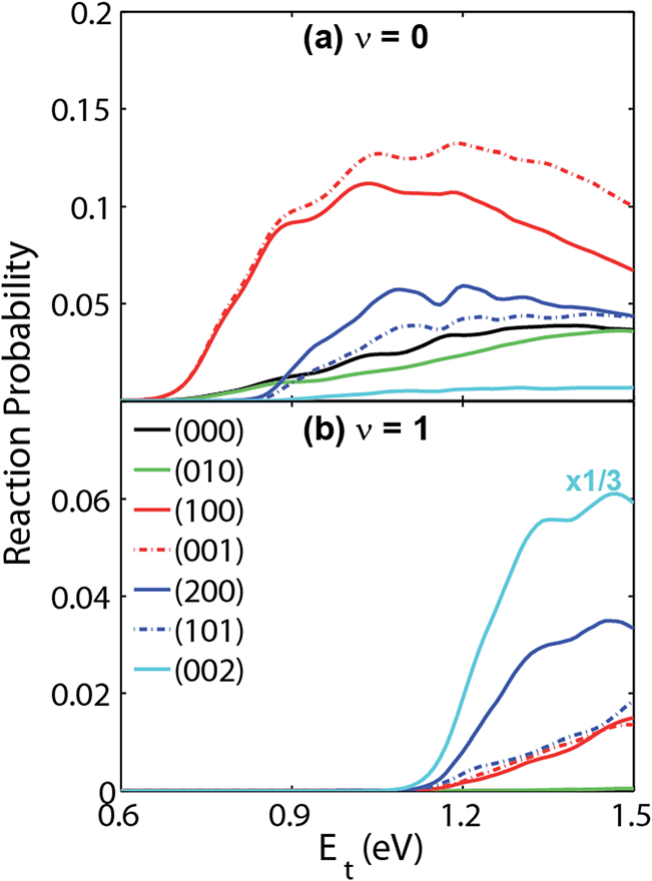
Vibrational state distribution (*J* = 0) of H_2_O in various normal mode states produced by reaction H_2_ + OH → H + H_2_O. When OH is in the ground vibrational state (a), the H_2_O product is dominated by the (100) and (001) state. However, the H_2_O product from the H_2_ + OH (*v* = 1) reaction (b) is dominated by the (002) state. Adapted from ref. 29 with permission.

**Fig. 4:**
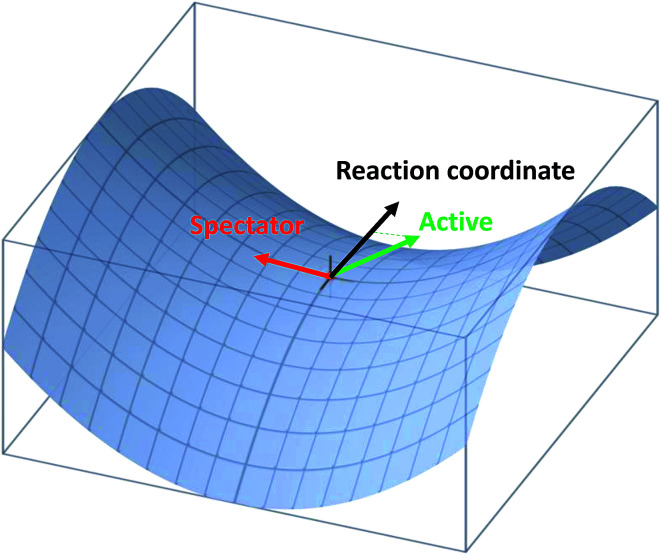
Illustration of the projection of an active and spectator modes onto the reaction coordinate at the transition state.

Very recently, the transition-state control of the H_2_ + OH reaction, the reverse reaction of H + H_2_O, was investigated at the state-to-state level.^78^ This reaction produces stretching excited H_2_O, mostly with one stretching quantum at low energies, which can be understood as a result of the fact that the H_2_O stretching modes are strongly coupled with the reaction coordinate at the transition state. Indeed, the symmetric and antisymmetric stretching modes of the H_2_O products are found to have roughly the same population, consistent with the local-mode picture discussed above. More interestingly, the H_2_O product is dominated by the (002) state when the OH reactant is in its first excited vibrational state, as shown in Fig. 3. A local-mode analysis revealed that the (002) normal mode state corresponds to the |11〉 local mode state, in which the H_2_O product contains one quantum of excitation in each OH bond. One OH quantum is apparently from the excited spectator OH moiety, while the other derives from the strong coupling of the H_2_O stretching vibrations with the reaction coordinate at the transition state. An intriguing observation here is that the product state distribution of this reaction depends sensitively on the vibrational excitation of the OH reactant. This state-to-state mode specificity appears to be quite different from the “memory loss” effect in the reverse reaction (H + H_2_O), as discussed above. The key to reconcile these two extremes is to recognize that the non-reactive OH moiety is a spectator in the reverse reaction, which has near-zero coupling with the reaction coordinate at the transition state. As a result, energy deposited into this mode is unlikely to leak out to the reaction coordinate, leading to no enhancement of reactivity. In other words, its energy is sequestered during the reaction. On the other hand, the water stretching vibrations are active modes in the forward reaction that have significant coupling with the reaction coordinate at the transition state, resulting in facile energy flow from these modes to the reaction coordinate and enhanced reactivity. In the meantime, the same energy flow depletes the energy in these modes, resulting in the loss of memory. In other words, the “memory loss” effect suggests that the product energy disposal is almost completely determined by the transition state, as advocated by the SVP model, and illustrated in Fig. 4.

Interestingly, the manner in which the transition state regulates the reaction can be visualized by the transition-state wave packet method.^35,79^ It has been shown that quantum states in the transition-state region of the PES, represented in that method by the eigenstates of the thermal flux operator, controls the reaction flux. In the case of X + H_2_O reaction, recent quantum state-to-state calculations have shown that such control is exercised in a coherent manner, in which several such thermal flux eigenstates act concertedly to regulate the flow of the reaction flux.^80,81^ Later work further indicates that OH excited thermal flux eigenstates are responsible for the energy sequestration of vibrational energy in the spectator OH mode in such reactions.^78^

The H/F + H_2_O → H_2_/HF + OH reactions have also been probed *via* transition-state spectroscopy. To this end, the stable species H_3_O^−^ or FH_2_O^−^ are photodetached to produce a wave packet near the transition state of the corresponding reaction. Very different wave packet dynamics were observed for these two reactions. In the H_3_O^−^ case, the dynamics are very fast and the photoelectron spectrum has no structure.^82,83^ On the other hand, the photoelectron spectrum of FH_2_O^−^ is highly structured, featuring both reactive and non-reactive Feshbach resonances.^84,85^

**Fig. 5:**
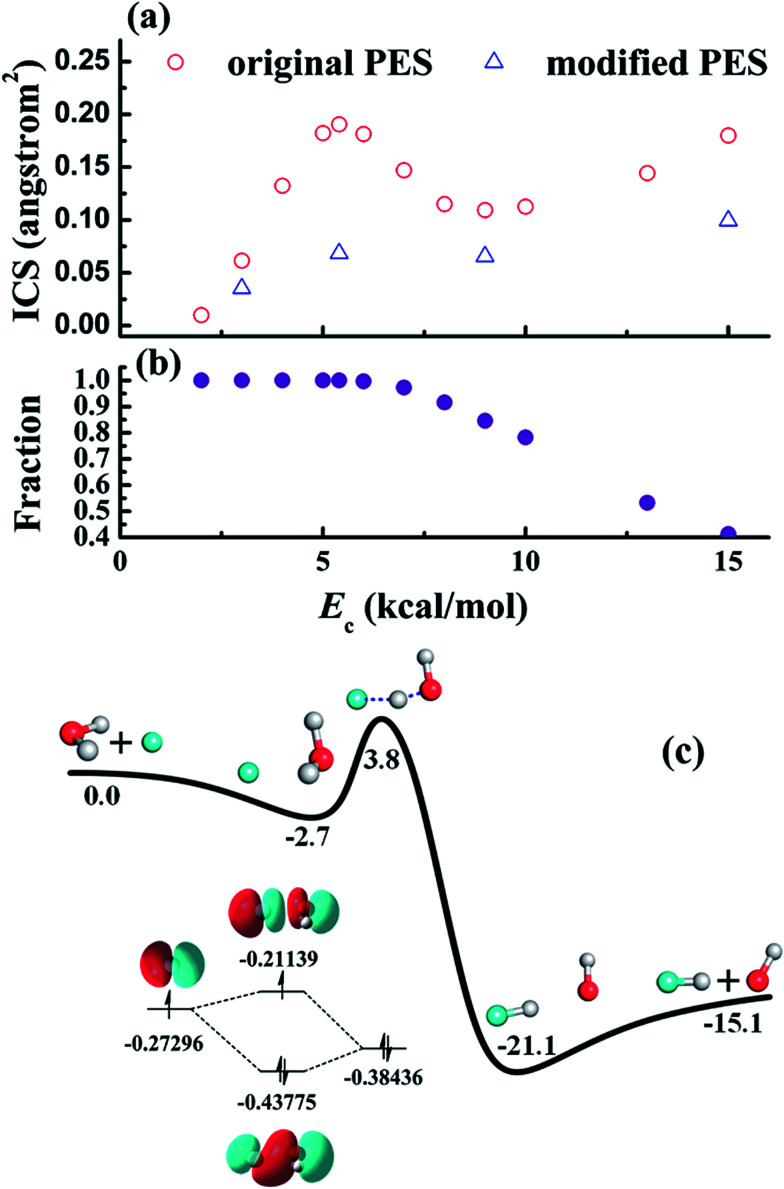
(a) Calculated reaction cross-sections of the F + H_2_O → HF + OH reaction based on an *ab initio* PES (original) and a modified PES (modified) in which the F–H_2_O well is artificially removed. The peak near 6 kcal mol^−1^ is thus attributed to the F–H_2_O well, through which the majority of the reactive trajectories are steered towards the reaction barrier, as shown in (b). The strong stereodynamic forces in this reaction is due to the F–H_2_O pre-reaction well, shown in (c), which has a hemi-bond character. Reproduced with permission from ref. 86 and 87.

It is important to realize that the transition-state control of the reactivity is not always absolute. Modifications of such control can be induced by either potential or kinetic sources. The former is facilitated by features on PESs. While the H + H_2_O ↔ H_2_ + OH reaction has very shallow pre- and post-reaction barriers, the other X + H_2_O reactions have significant wells along the reaction pathway. The pre-reaction well might exert strong forces on the reactants, whereas the post-reaction well could impact the final state distribution of the products. An interesting case is the pre-reaction well of the F + H_2_O reaction, which is relatively deep due to its hemi-bond (two-center, three-electron bond) character, as shown in Fig. 5.^86^ For this low barrier reaction, the stereodynamic forces at low collision energies are strong enough to cause a significant enhancement of the reactivity.^87^ This modification has also a quantum mechanical character, manifesting in the form of tunneling.^88^ Similar stereodynamic forces have been seen in many other systems, including Cl + HD^89^ and F + CHD_3_ reactions.^90,91^

### B. X + CH_4_ ↔ HX + CH_3_ reactions

This series of six-atom reactions, with X = H, O(^3^P), F, and Cl, has become a benchmark to advance our understanding of polyatomic chemical reactivity not only because of their fundamental interests in offering a wide range of diverse PES topographies, but also for their important roles in combustion, atmospheric, and astrophysical chemistry.^12,23,38^ The reaction of H + CH_4_ exhibits a high barrier of 14.7 kcal mol^−1^ with a small endothermicity of 2.8 kcal mol^−1^. At the transition state the H–H distance is slightly elongated from the equilibrium value in H_2_, while the C–H distance is significantly stretched from the bond length in methane.^92–95^ By Hammond's postulate^60^ the transition-state structure is product-like, and by the Polanyi rules^8^ the reaction is characterized by a late barrier. Upon one quantum excitation of the asymmetric stretching mode (*v*_3_ = 1) of CH_4_, a rate enhancement of ∼3 was found experimentally in H + CH_4_ at the same collision energy,^96^ consistent with the Polanyi rule for a late barrier reaction. Several theoretical calculations have since confirmed this vibrational effect on reactivity.^97–101^ The SVP model also indicates a moderate coupling of the *v*_3_ mode with the reaction coordinate at the transition state, and thus a vibrational enhancement.^38^ This vibrational effect underscores the transition-state control of reactivity. The SVP model further predicts a slightly stronger coupling for the symmetric stretching mode *v*_1_;^38^ but experimental confirmation has not yet been reported for this mode. Bond selectivity has also been observed^102^ and confirmed theoretically^103,104^ in the H + CHD_3_ reaction, in which the local C–H stretching mode (*v*_1_ = 1) excitation in CHD_3_ promotes the H-atom abstraction channel. This bond selectivity can readily be rationalized by the SVP model as the *v*_1_ mode is essentially the reaction coordinate.^38^

The reverse reaction H_2_ + CH_3_ has been studied theoretically.^105,106^ The results from reduced-dimensional QD calculations indicated that the H_2_ stretching and CH_3_ umbrella modes, as well as the translational energy, strongly promote the reaction, while the CH_3_ symmetric stretching mode behaves as a spectator exerting little effect. The observed mode specificity is confirmed by full-dimensional quasi-classical trajectory (QCT) calculations.^105^ Again, the SVP model provides rationalization of the mode specificity in terms of the coupling strengths of reactant modes with the reaction coordinate at the transition states.^105^

**Fig. 6:**
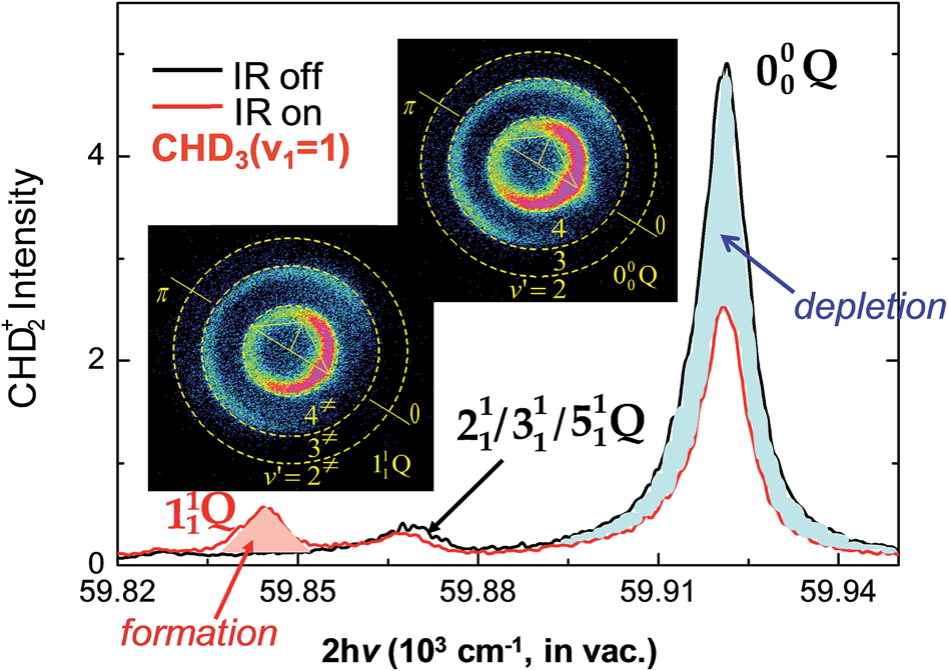
Two normalized REMPI spectra of the probed CHD_2_ products, with IR-on (red line) and IR-off (black), at *E*_c_ = 3.6 kcal mol^−1^. A significant reduction of the signals for the origin band 0^0^_0_ (blue-shaded area) and the formation of a new band 1^1^_1_ (red-shaded area) are observed. Two product images, both with IR-on, are shown for probing the 1^1^_1_ Q (left-lower) and 0^0^_0_ Q (right-upper) bands, respectively. Superimposed on the images are the scattering directions; the 0° angle refers to the initial CHD_3_ beam direction in the center-of-mass frame (modified with permission from ref. 90).

In contrast to the above H + CH_4_ case, the reaction of F + CH_4_ is highly exothermic by 31.8 kcal mol^−1^ with a low barrier of about 0.7 kcal mol^−1^ (with zero-point-energy corrected) in the entrance valley,^107^*i.e.*, an early barrier. According to the Polanyi rules,^8^ the initial translation energy should be more efficacious than the vibrational excitation of methane in promoting the reaction rate. In addition, with a reactant-like transition-state structure, the vibrational motion of reactant would have little coupling to the reaction coordinate, as also indicated by the SVP model.^38^ Hence, the vibrational modes should behave as a spectator in F + methane, as such one might have expected little reactivity change upon the vibrational excitation of methane. Counter-intuitively, the excitation of the C–H stretching mode in CHD_3_ was found to inhibit the C–H bond rupture leading to the HF + CD_3_ product in F + CHD_3_ (*v*_1_ = 1). As shown in Fig. 6,^90^ the IR-on REMPI spectrum clearly displays a negative impact to the formation of the dominant CHD_2_ (*v* = 0) products, which demonstrates, from eqn (3), that the stretch-excited reactivity should be smaller than the ground state reactivity. In fact, significant depletions of the product REMPI signals were observed for all, including the CH_3_ + DF channel,^90,108,109^ but the CHD_2_ (*v*_1_ = 1) state. The latter can be regarded as the spectator when the F-atom abstracts one of the unexcited D-atoms, leaving the initially deposited vibration energy in the excited C–H bond of the CHD_2_ products. Similar inhibitory results was reported in the F + CH_4_ (*v*_3_ = 1) reaction.^110^

This surprising effect was speculated as the result of the stereodynamic forces exerting on the approaching reactants by the pre-reaction van der Waals (vdW) wells. The experimental conjecture was later borne out in a QCT calculation on an accurate PES,^91^ which highlights the possibility that anisotropic forces modifies the transition-state control of reaction dynamics for low, early-barrier reactions. However, more recent theoretical investigations, also by QCT but on different PESs in the entrance valley, suggested no inhibitory effect by CHD_3_ (*v*_1_ = 1).^111,112^ The source of the theory-experiment discrepancy is unclear at present. On the experimental side, it could arise from some other excited methyl products, which are inaccessible to the current REMPI detection method and yet with much higher abundances than those states being probed thus far. On the theory side, as other experimental evidence strongly suggests a resonance-mediated pathway in F + methane,^113,114^ the QCT calculations may not capture the quantum nature of this reaction. Alternatively, theory and experiment may refer to subtly different quantities. Whereas the experiment results correspond to the relative reactivity of F + CHD_3_ (*v*_1_ = 1, |*J*, *K*〉 = |2, 0〉 and |2, ±1〉) to that of F + CHD_3_ (*v* = 0, *T*_rot_ ∼ 9 K), the theory refers to the single rotational |*J*, *K*〉 = |0, 0〉 state for both CHD_3_ (*v*_1_ = 1) and CHD_3_ (*v* = 0). As will be discussed below in the Cl + CHD_3_ (*v*_1_ = 1, |*J*, *K*〉) reaction, the initially selected rotational |*J*, *K*〉-states can result in profound effects on reactivity. More studies, both experiment and theory, are needed to reconcile the discrepancy.

It is interesting to note that in the above F + H_2_O case, which is also very exothermic with a low barrier and a relatively deep pre-reaction well, the stretching excitation of water is predicted to significantly enhance the reactivity,^71^ in opposite to the F + CH_4_ reaction. In both cases, the transition-state control of reactivity is strongly modulated by the long-range anisotropic interactions in the entrance valley, yet resulting in vastly different outcomes. The effects of bending excitation of CD_4_ and CHD_3_ in reaction with F-atom have also been studied,^115–117^ in which both experiment and theory (QCT) show that bending excitation activates the reactivity at low collision energies and become inactivated at higher collision energies. Experimental results on both the integral and differential cross sections further suggested signature for quantum resonances in bend-excited reactions, which await further theoretical investigations.^116,117^

Unlike the late barrier in the H + CH_4_ reaction and early barrier in the F + CH_4_ reaction, the reaction between O(^3^P) and CH_4_ presents an interesting case in which the transition state has a central location with a barrier height of ∼14 kcal mol^−1^.^118^ The Polanyi rules thus provide little guidance on the mode specificity in such reactions. Reduced-dimensional QD and full-dimensional QCT calculations revealed significant mode specificity,^119,120^ which can be rationalized by the SVP model.^38^ The theoretical results are found to be consistent with the experimental data.^121,122^

The hydrogen abstraction reaction between Cl and methane is perhaps one of the most studied polyatomic reactions in both experiment and theory. It is slightly endothermic by 1.2 kcal mol^−1^ and with a late (adiabatic) barrier height of 3.4 kcal mol^−1^.^123^ In accord with the Polanyi rules as well as the SVP model, both experiments and theoretical calculations indicate reactivity promotion by stretching excitations of methane reactants. A number of reviews have been devoted to the mode specificity and bond selectivity of this reaction,^9–14^ to which the interested readers are referred. More recent studies have turned the attention to its stereochemical aspects.^10,40^ Two complementary approaches have been taken to elucidate the effects of the collisional geometry on chemical reactivity: by rotational state selectivity of the vibrationally excited methane and by prealigning the excited methane in space for a given rovibrationally selected state. These studies allow unprecedented control of collision events, eliminating averaging in the rotational and orientational levels. They shed valuable light on the “chemical shape”^124^ of the reactant molecules as they react with their collisional partners.

**Fig. 7:**
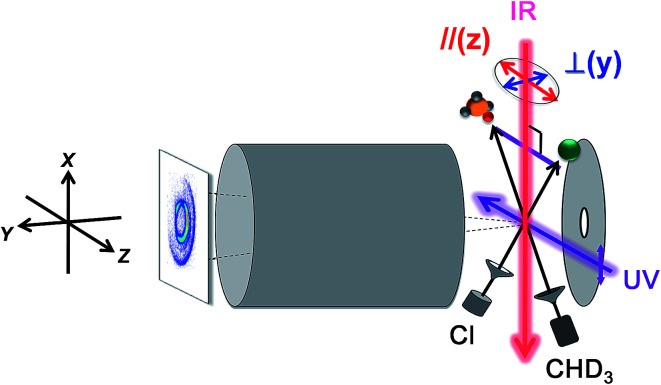
Experimental setup for state-selected, aligned molecular beam scattering experiments. All four beams (two molecular beams, IR-pump, and UV-probe lasers) lie in the *xz* plane. The REMPI-tagged ions are velocity-mapped and guided along the *y*-direction. A linearly polarized IR laser directed perpendicularly to the relative velocity vector of the two molecular beams prepares the vibrationally excited CHD_3_ at the scattering center. Reagent alignment is controlled by IR laser polarization direction, “//” refers to an end-on attack and “⊥” to a side-on approach. Time-sliced velocity-map image, shown at the left, reveals the alignment effects on product pair-correlated distribution (modified with permission from ref. 125).

**Fig. 8:**
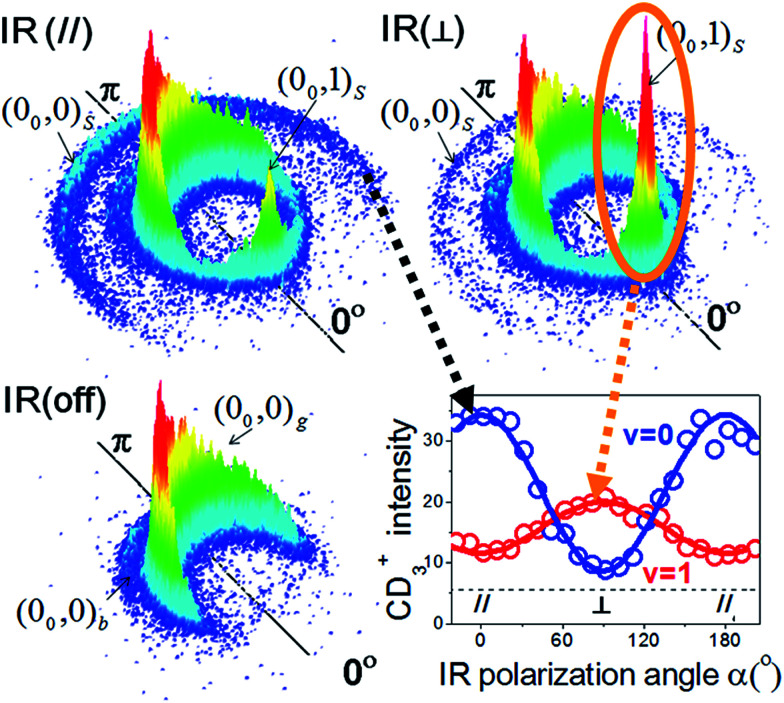
Three CD_3_ (*v* = 0) product images with a superimposed axis indicating the scattering direction; the 0° angle refers to the initial CHD_3_ beam direction in the center-of-mass frame of Cl + CHD_3_ reaction. The distinct ring-like features are dictated by the conservation of energy and momentum. The pair-correlated labeling is defined as follows: the numbers in the parentheses denote the (*v*_CD_3__, *v*_HCl_) products; the outer subscript indicates the CHD_3_ reactant state (“g” for ground state, “b” for bend-excited state, and “s” for the stretch-excited state). The lower-right panel shows the polarization-angle dependence of the probed signals from the stretch-excited reaction. The *v* = 0 data correspond to the outer-ring feature labeled (0_0_, 0)_s_; for the *v* = 1 data, only the forward signals for (0_0_, 1)_s_, as encircled, are counted (modified with permission from ref. 125).

Fig. 7 illustrates an experimental setup for measuring the reactivity effect of aligned CHD_3_ (*v*_1_ = 1, |*J*, *K*〉 = |1, 0〉).^125^ As depicted, four beams (two molecular beams, IR-excitation and UV-probe lasers) lie in the same *x*–*z* plane, and the initial relative velocity can be arranged being perpendicular (parallel) to the IR (UV) laser. Because the *v*_1_ = 1 ← 0 transition is a parallel band with the transition dipole moment lying along the C–H bond, varying the linear polarization of the IR laser effectively changes the direction of the excited C–H bond with respect to the approaching Cl-atom in the collision frame. Fig. 8 presents the raw data. The IR-off image represents the ground state reaction, yielding a pair of CD_3_ (*v* = 0) + HCl (*v* = 0) products, *i.e.* the (0_0_, 0)_g_ state pair (the subscript “g” denotes the ground-state reaction). Two IR-on images correspond to the CD_3_ (*v* = 0) signals from the stretch-excited reaction (plus the residual ground state signals from the unexcited CHD_3_), differing only by the IR-polarization directions. The distinct patterns of the new features in the two IR-on images signify a strong stereodynamic effect in the Cl + CHD_3_ (*v*_1_ = 1, |*J*, *K*〉 = |1, 0〉) reaction. The right-lower panel of Fig. 8 shows the IR-polarization dependencies of the two product pairs, (0_0_, 0)_s_ and (0_0_, 1)_s_, from the stretch-excited reactions. Interestingly, the two product state pairs display a striking out-of-phase oscillation. A more detailed analysis offers direct experimental evidence for a collinear Cl–H–C transition-state structure and allows a 3D visualization of how the chemical transformation is taking place.^126–128^

Moreover, the experiment also showed a maximal alignment effects allowed by quantum mechanics.^127^ The implication is that the asymptotically aligned CHD_3_ (*v*_1_ = 1) reactant must retain its directional properties en route to the reaction barrier; otherwise, a weaker polarization dependency should have been observed. This is quite intriguing in that the interaction between Cl and CHD_3_ also exhibits vdW wells in the entrance valley with the well depths deeper than the above F + CHD_3_ system. Yet, a significant reorientation of the approaching F-atom was surmised in the latter reaction as discussed above. In that regard, we note that the vibrationally enhanced reactivity has also been observed in O(^3^P) + CHD_3_ (*v*_1_ = 1) by enlarging the cone of the reactive acceptance due to the focusing effects of the long-range anisotropic interactions en route to the barrier.^121^ The three seemly analogous reactions all have similar vdW topographies in the entrance valley, yet display vastly contrasting stereodynamic behaviors. Will it be subtly related to the diverse barrier locations or other features in PESs? The challenge is to gain deeper insights into these perplexing findings and to comprehend the underlying origin, which remains a challenge for theory.

**Fig. 9:**
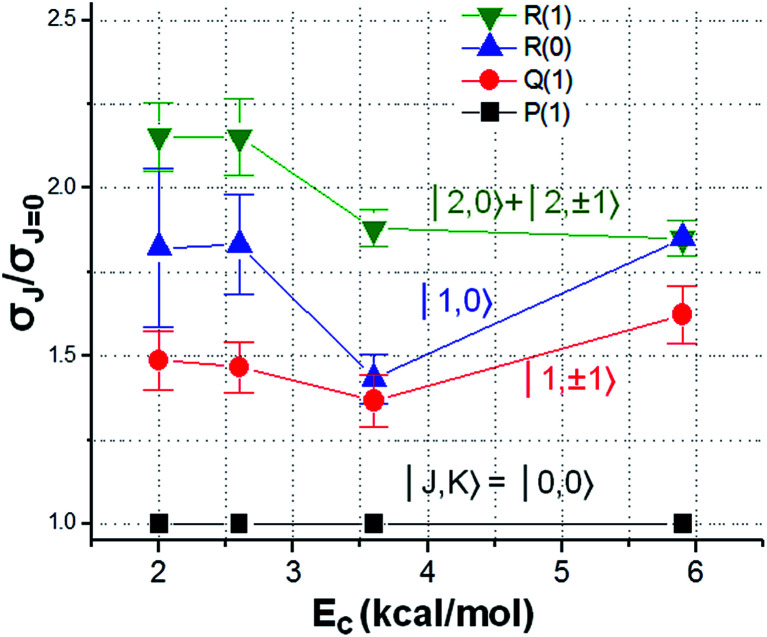
Dependency of relative reactivity on the rotational states of vibrationally excited CHD_3_ (*v*_1_ = 1) reactants in reaction with Cl atom. At each *E*_c_, the reactivity of the rotational ground state is set at unity (modified with permission from ref. 129).

An alternative approach to elucidate the geometric dependency of reactivity is to examine the rotational mode specificity. Exemplified in Fig. 9 is the relative reactivity for the ro-vibrationally selected CHD_3_ (*v*_1_ = 1, |*J*, *K*〉) reactants with Cl-atoms.^129,130^ A clear *J*- and *K*-dependency is revealed: a rotating CHD_3_ (*v*_1_ = 1) reactant promotes the reactivity, and for a given *J* the tumbling rotation (*K* = 0) yields higher reactivity than the spinning rotation (|*K*| = *J*). Concurrent QCT and reduced dimensionality QD calculations confirmed the observed trends and offered an intuitively appealing explanation: the range of the attack angles near the barrier opens up (*i.e.*, the bending potential at the transition state becomes softer) for the rotating reactants.^110^ It is worth noting that the initial rotational energy difference is very small for those low *J*/*K* states; for example, *E*_*J*=2_ is merely 0.055 kcal mol^−1^ above the rotationless state. Hence, the observed rotational enhancement factor of ∼2 is quite significant. In particular, it is about the same magnitude, on the equivalent amount of total energy, as the reported vibrational enhancement factor in Cl + CHD_3_ (*v*_1_ = 1).^131,132^ As the experimental vibrational factors reported in literatures often are rotationally ill-defined or in some cases for the *J* = 2 state, it becomes somewhat ambiguous, in view of the rotational mode specificity illustrated here, as to how to untangle the “pure” vibrational enhancement from a collective effect of ro-vibrational excitation. This serves as a cautionary note for theory-experiment comparison and for future studies.

## V. Implications in larger reactions

The principles discussed above for relatively small reactive systems are applicable to more complex reactions. One such example is the 1,3 dipolar cycloaddition reaction between a 1,3 dipole and a dipolarophile to form a five-membered ring, *via* a concerted transition state.^133^ This classical organic reaction is a key step in click chemistry.^134^ Recently, Houk and coworkers have carried out extensive direct dynamics calculations, starting the trajectories from the transition state towards the reactant channel.^135,136^ They found that the bending vibration of the 1,3 dipoles is always excited in these trajectories. Based on microscopic reversibility, it was argued that the bending excitation in the 1,3 dipoles enhances reactivity of these reactions, which is consistent with the fact that these reactions require heating. Interestingly, the involvement of the 1,3 dipole bending vibration in such reactions can actually be predicted by the SVP model, without the expensive direct dynamics calculations. In a recent study,^137^ we have shown that reactant bending modes have large overlaps with the reaction coordinate at the transition state, in support of the conclusion of Houk and coworkers. In addition, the SVP model also predicts enhancement effects for several other reactant modes, which are to be confirmed.

This example suggests that the transition state exerts strong control of dynamics even in organic reactions involving large molecules.^138^ One distinct advantage of the SVP model is that its predictions only require information on the transition state as well as on the reactants. The reaction coordinate can be readily determined using any *ab initio* method, without the complete PES, which is increasingly more difficult to obtain as the system becomes larger. As a result, this model is particularly useful for ever more complex reactions.

## VI. Conclusions

Recent advances in quantum state resolved reaction dynamics have revealed an unprecedented level of detail for bimolecular reactions. Experimentally, it has now become possible to selectively excite the reactants into a single quantum state and to align them, in addition to measuring the product state distributions with the most exquisite detail. Theoretically, accurate PESs can now be constructed based on a large number of *ab initio* points, upon which QD and QCT calculations can be carried out. The accumulation of data in both theory and experiment has led to a much more in-depth understanding of how the transition-state controls the reactivity. The examples discussed in this perspective have demonstrated convincingly that such a control lies at the heart of the observed mode specificity and bond selectivity. Our knowledge on these phenomena is best illustrated by a simple transition-state based construct, such as the SVP model, in which the ability of a particular reactant mode in promoting the reaction is attributed to the projection of the corresponding normal mode vector onto the reaction coordinate at the transition state. The SVP model, which can be considered as a generalization of the venerable Polanyi rules, particularly for reactions involving polyatomic molecules, has been shown to be generally successful in its predictions, as long as the sudden approximation, namely the assumption that the collision time is much shorter than the IVR rate, is valid. While the transition-state control of reactivity is in general quite dominant, evidence has also been presented for the modifications of the transition state control of reactivity. These modifications stem from features on the PES that become non-negligible under specific conditions. The most important point is perhaps that the lessons learned in reactions involving small molecules can be extended to larger reactive systems, of which the transition-state control of their reactivity has seldom been explored.

## References

[cit1] Eyring H. (1935). J. Chem. Phys..

[cit2] Wigner E. (1937). J. Chem. Phys..

[cit3] Evans M. G., Polanyi M. (1939). Trans. Faraday Soc..

[cit4] Steinfeld J. I. , Francisco J. S. and Hase W. L., Chemical Kinetics and Dynamics, Prentice Hall, Englewood Cliffs, NJ, 1989

[cit5] Truhlar D. G., Garrett B. C., Klippenstein S. J. (1996). J. Phys. Chem..

[cit6] Polanyi J. C., Zewail A. H. (1995). Acc. Chem. Res..

[cit7] Levine R. D. , Molecular Reaction Dynamics, Cambridge University Press, Cambridge, 2005

[cit8] Polanyi J. C. (1987). Science.

[cit9] Guo H., Jiang B. (2014). Acc. Chem. Res..

[cit10] Liu K. (2015). J. Chem. Phys..

[cit11] Zhang D. H., Guo H. (2016). Annu. Rev. Phys. Chem..

[cit12] Liu K. (2016). Annu. Rev. Phys. Chem..

[cit13] Crim F. F. (1999). Acc. Chem. Res..

[cit14] Crim F. F. (2008). Proc. Natl. Acad. Sci. U. S. A..

[cit15] Zare R. N. (1998). Science.

[cit16] Liu K. (2001). Annu. Rev. Phys. Chem..

[cit17] Balucani N., Capozza G., Leonori F., Segoloni E., Casavecchia P. (2006). Int. Rev. Phys. Chem..

[cit18] Liu K. (2007). Phys. Chem. Chem. Phys..

[cit19] Yang X. (2007). Annu. Rev. Phys. Chem..

[cit20] Althorpe S. C., Clary D. C. (2003). Annu. Rev. Phys. Chem..

[cit21] Aoiz F. J., Bañares L., Herrero V. J. (2005). Int. Rev. Phys. Chem..

[cit22] Guo H. (2012). Int. Rev. Phys. Chem..

[cit23] Czakó G., Bowman J. M. (2014). J. Phys. Chem. A.

[cit24] Li J., Jiang B., Song H., Ma J., Zhao B., Dawes R., Guo H. (2015). J. Phys. Chem. A.

[cit25] Fernandez-Alonso F., Zare R. N. (2002). Annu. Rev. Phys. Chem..

[cit26] Liu K. (2012). Adv. Chem. Phys..

[cit27] Sun Z. , Zhao B., Liu S. and Zhang D. H., in Molecular Quantum Dynamics, From Theory to Application, ed. F. Gatti, Springer, Heidelberg, 2014

[cit28] Jiang B., Guo H. (2013). J. Chem. Phys..

[cit29] Jiang B., Guo H. (2013). J. Am. Chem. Soc..

[cit30] Schatz G. C., Ross J. (1977). J. Chem. Phys..

[cit31] Wang D., Bowman J. M. (1993). Chem. Phys. Lett..

[cit32] Havalee U., Shapiro M. (1976). J. Chem. Phys..

[cit33] Schinke R. , Photodissociation Dynamics, Cambridge University Press, Cambridge, 1993

[cit34] Gustafsson M., Skodje R. T. (2006). J. Chem. Phys..

[cit35] Welsch R., Huarte-Larrañaga F., Manthe U. (2012). J. Chem. Phys..

[cit36] Wang Y., Bowman J. M. (2013). J. Chem. Phys..

[cit37] Siebrand W., Smedarchina Z., Fernández-Ramos A. (2013). J. Chem. Phys..

[cit38] Jiang B., Guo H. (2014). J. Chin. Chem. Soc..

[cit39] Miller W. H., Handy N. C., Adams J. E. (1980). J. Chem. Phys..

[cit40] Simpson W. R., Orr-Ewing A. J., Rakitzis T. P., Kandel S. A., Zare R. N. (1995). J. Chem. Phys..

[cit41] Lin J. J., Zhou J., Shiu W., Liu K. (2003). Rev. Sci. Instrum..

[cit42] Chandler D. W., Houston P. L. (1987). J. Chem. Phys..

[cit43] Eppink A. T. J. B., Parker D. H. (1997). Rev. Sci. Instrum..

[cit44] Gebhardt C. R., Rakitzis T. P., Samartzis P. C., Ladopoulos V., Kitsopoulos T. N. (2001). Rev. Sci. Instrum..

[cit45] Townsend D., Minitti M. P., Suits A. G. (2003). Rev. Sci. Instrum..

[cit46] Lin J. J., Zhou J., Shiu W., Liu K. (2003). Science.

[cit47] Zhou J. G., Lin J. J., Shiu W. C., Liu K. (2006). Phys. Chem. Chem. Phys..

[cit48] Sonnenfroh D. M., Liu K. (1991). Chem. Phys. Lett..

[cit49] Proctor D. L., Davis H. F. (2008). Proc. Natl. Acad. Sci. U. S. A..

[cit50] Yan S., Wu Y.-T., Liu K. (2007). Phys. Chem. Chem. Phys..

[cit51] Riedel J., Yan S., Kawamata H., Liu K. (2008). Rev. Sci. Instrum..

[cit52] Cheng Y., Pan H., Wang F., Liu K. (2014). Phys. Chem. Chem. Phys..

[cit53] Shiozaki T., Werner H.-J. (2013). Mol. Phys..

[cit54] Xiao C., Xu X., Liu S., Wang T., Dong W., Yang T., Sun Z., Dai D., Xu X., Zhang D. H., Yang X. (2011). Science.

[cit55] Schatz G. C. (1981). J. Chem. Phys..

[cit56] Sinha A., Hsiao M. C., Crim F. F. (1991). J. Chem. Phys..

[cit57] Sinha A., Thoemke J. D., Crim F. F. (1992). J. Chem. Phys..

[cit58] Bronikowski M. J., Simpson W. R., Zare R. N. (1993). J. Phys. Chem..

[cit59] Thoemke J. D., Pfeiffer J. M., Metz R. B., Crim F. F. (1995). J. Phys. Chem..

[cit60] Hammond G. S. (1955). J. Am. Chem. Soc..

[cit61] Child M. S., Halonen L. (1984). Adv. Chem. Phys..

[cit62] Zhang D. H., Collins M. A., Lee S.-Y. (2000). Science.

[cit63] Fu B., Zhang D. H. (2013). J. Chem. Phys..

[cit64] Song H., Guo H. (2015). J. Phys. Chem. A.

[cit65] Sinha A., Hsiao M. C., Crim F. F. (1990). J. Chem. Phys..

[cit66] Bronikowski M. J., Simpson W. R., Zare R. N. (1993). J. Phys. Chem..

[cit67] Zhang D. H., Light J. C. (1997). J. Chem. Soc., Faraday Trans..

[cit68] Li J., Song H., Guo H. (2015). Phys. Chem. Chem. Phys..

[cit69] Fu B., Zhang D. H. (2015). J. Chem. Phys..

[cit70] Song H., Lee S.-Y., Lu Y., Guo H. (2015). J. Phys. Chem. A.

[cit71] Li J., Jiang B., Guo H. (2013). J. Am. Chem. Soc..

[cit72] Song H., Guo H. (2015). J. Phys. Chem. A.

[cit73] Zolot A. M., Nesbitt D. J. (2008). J. Chem. Phys..

[cit74] Li J., Dawes R., Guo H. (2012). J. Chem. Phys..

[cit75] Li J., Jiang B., Guo H. (2013). J. Chem. Phys..

[cit76] Welsch R., Manthe U. (2015). J. Phys. Chem. Lett..

[cit77] Liu S., Zhang D. H. (2016). Chem. Sci..

[cit78] Zhao B., Sun Z., Guo H. (2015). J. Am. Chem. Soc..

[cit79] Zhao B., Sun Z., Guo H. (2014). J. Chem. Phys..

[cit80] Zhao B., Sun Z., Guo H. (2014). J. Chem. Phys..

[cit81] Zhao B., Sun Z., Guo H. (2015). J. Chem. Phys..

[cit82] de Beer E., Kim E. H., Neumark D. M., Gunion R. F., Lineberger W. C. (1995). J. Phys. Chem..

[cit83] Zhang D. H., Yang M., Collins M. A., Lee S.-Y. (2002). Proc. Natl. Acad. Sci. U. S. A..

[cit84] Otto R., Ma J., Ray A. W., Daluz J. S., Li J., Guo H., Continetti R. E. (2014). Science.

[cit85] Ma J., Guo H. (2015). J. Phys. Chem. Lett..

[cit86] Li J., Li Y., Guo H. (2013). J. Chem. Phys..

[cit87] Li J., Jiang B., Guo H. (2013). Chem. Sci..

[cit88] Zhao B., Guo H. (2015). J. Phys. Chem. Lett..

[cit89] Skouteris D., Castillo J. F., Manolopoulos D. E. (2000). Comput. Phys. Commun..

[cit90] Zhang W., Kawamata H., Liu K. (2009). Science.

[cit91] Czakó G., Bowman J. M. (2009). J. Am. Chem. Soc..

[cit92] Zhang X., Braams B. J., Bowman J. M. (2006). J. Chem. Phys..

[cit93] Wu T., Werner H.-J., Manthe U. (2006). J. Chem. Phys..

[cit94] Corchado J. C., Bravo J. L., Espinosa-García J. (2009). J. Chem. Phys..

[cit95] Zhou Y., Fu B., Wang C., Collins M. A., Zhang D. H. (2011). J. Chem. Phys..

[cit96] Camden J. P., Bechtel H. A., Brown D. J. A., Zare R. N. (2005). J. Chem. Phys..

[cit97] Wang M. L., Zhang J. Z. H. (2002). J. Chem. Phys..

[cit98] Xie Z., Bowman J. M., Zhang X. (2006). J. Chem. Phys..

[cit99] Rangel C., Corchado J. C., Espinosa-García J. (2006). J. Phys. Chem. A.

[cit100] Schiffel G., Manthe U. (2010). J. Chem. Phys..

[cit101] Liu R., Xiong H., Yang M. (2012). J. Chem. Phys..

[cit102] Camden J. P., Bechtel H. A., Brown D. J. A., Zare R. N. (2006). J. Chem. Phys..

[cit103] Xie Z., Bowman J. M. (2006). Chem. Phys. Lett..

[cit104] Zhou Y., Wang C., Zhang D. H. (2011). J. Chem. Phys..

[cit105] Wang Y., Li J., Chen L., Lu Y., Yang M., Guo H. (2015). J. Chem. Phys..

[cit106] Zhang Z., Chen J., Yang M., Zhang D. H. (2015). J. Phys. Chem. A.

[cit107] Czakó G., Shepler B. C., Braams B. J., Bowman J. M. (2009). J. Chem. Phys..

[cit108] Yang J., Zhang D., Jiang B., Dai D., Wu G., Zhang D., Yang X. (2014). J. Phys. Chem. Lett..

[cit109] Yang J., Zhang D., Chen Z., Blauert F., Jiang B., Dai D., Wu G., Zhang D., Yang X. (2015). J. Chem. Phys..

[cit110] Kawamata H., Zhang W. Q., Liu K. (2012). Faraday Discuss..

[cit111] Palma J., Manthe U. (2015). J. Phys. Chem. A.

[cit112] Espinosa-Garcia J. (2016). J. Phys. Chem. A.

[cit113] Zhou J., Lin J. J., Liu K. (2004). J. Chem. Phys..

[cit114] Zhou J., Lin J. J., Liu K. (2010). Mol. Phys..

[cit115] Czakó G., Shuai Q. A., Liu K., Bowman J. M. (2010). J. Chem. Phys..

[cit116] Wang F., Liu K. (2011). J. Phys. Chem. Lett..

[cit117] Wang F., Liu K. (2013). J. Phys. Chem. A.

[cit118] Czakó G., Bowman J. M. (2012). Proc. Natl. Acad. Sci. U. S. A..

[cit119] Liu R., Yang M., Czakó G., Bowman J. M., Li J., Guo H. (2012). J. Phys. Chem. Lett..

[cit120] Czakó G., Liu R., Yang M., Bowman J. M., Guo H. (2013). J. Phys. Chem. A.

[cit121] Wang F., Liu K. (2010). Chem. Sci..

[cit122] Zhang J., Liu K. (2011). Chem.–Asian J..

[cit123] Czakó G., Bowman J. M. (2012). J. Chem. Phys..

[cit124] Levine R. D. (1990). J. Phys. Chem..

[cit125] Wang F., Lin J.-S., Liu K. (2011). Science.

[cit126] Wang F., Liu K. (2013). Chin. J. Chem. Phys..

[cit127] Wang F., Liu K., Rakitzis T. P. (2012). Nat. Chem..

[cit128] Wang F., Lin J.-S., Liu K. (2014). J. Chem. Phys..

[cit129] Liu R., Wang F., Jiang B., Czakó G., Yang M., Liu K., Guo H. (2014). J. Chem. Phys..

[cit130] Wang F., Pan H., Liu K. (2015). J. Phys. Chem. A.

[cit131] Yan S., Wu Y. T., Zhang B., Yue X.-F., Liu K. (2007). Science.

[cit132] Wang F., Lin J.-S., Cheng Y., Liu K. (2013). J. Phys. Chem. Lett..

[cit133] Gothelf K. V., Jørgensen K. A. (1998). Chem. Rev..

[cit134] Kolb H. C., Finn M. G., Sharpless K. B. (2001). Angew. Chem., Int. Ed..

[cit135] Xu L., Doubleday C. E., Houk K. N. (2009). Angew. Chem., Int. Ed..

[cit136] Xu L., Doubleday C. E., Houk K. N. (2010). J. Am. Chem. Soc..

[cit137] Li A., Guo H. (2015). Chem. Phys. Lett..

[cit138] Barnes G. L., Hase W. L. (2009). Nat. Chem..

